# Geometry Calibration of a Modular Stereo Cone-Beam X-ray CT System

**DOI:** 10.3390/jimaging7030054

**Published:** 2021-03-13

**Authors:** Van Nguyen, Joaquim G. Sanctorum, Sam Van Wassenbergh, Joris J. J. Dirckx, Jan Sijbers, Jan De Beenhouwer

**Affiliations:** 1 Imec—Vision Lab, Department of Physics, University of Antwerp, 2610 Antwerp, Belgium; jan.debeenhouwer@uantwerpen.be; 2 Biophysics and BioMedical Physics (BIMEF) Lab, University of Antwerp, 2020 Antwerp, Belgium; joaquim.sanctorum@uantwerpen.be (J.G.S.); joris.dirckx@uantwerpen.be (J.J.J.D.); 3 Functional Morphology Lab (FunMorph), University of Antwerp, 2610 Antwerp, Belgium; sam.vanwassenbergh@uantwerpen.be

**Keywords:** stereo cone-beam geometry, geometry calibration, modular X-ray system

## Abstract

Compared to single source systems, stereo X-ray CT systems allow acquiring projection data within a reduced amount of time, for an extended field-of-view, or for dual X-ray energies. To exploit the benefit of a dual X-ray system, its acquisition geometry needs to be calibrated. Unfortunately, in modular stereo X-ray CT setups, geometry misalignment occurs each time the setup is changed, which calls for an efficient calibration procedure. Although many studies have been dealing with geometry calibration of an X-ray CT system, little research targets the calibration of a dual cone-beam X-ray CT system. In this work, we present a phantom-based calibration procedure to accurately estimate the geometry of a stereo cone-beam X-ray CT system. With simulated as well as real experiments, it is shown that the calibration procedure can be used to accurately estimate the geometry of a modular stereo X-ray CT system thereby reducing the misalignment artifacts in the reconstruction volumes.

## 1. Introduction

Stereo X-ray cone-beam CT refers to an acquisition setting with two X-ray source/ detector pairs that are positioned at different viewing angles with respect to the target object. With such a setting, data can be acquired simultaneously from two, e.g., orthogonal, directions, or in dual-energy mode. The stereo X-ray systems are widely used in image guided radio therapy applications as they provide a fast acquisition and reduce the exposure of the patients to ionizing radiation [1,2,3]. Stereo acquisition also allows reconstructing 3D object motion from only a few X-ray radiographs, when combined with a pre-recorded CT volume [2,4,5]. A stereo circular cone-beam X-ray CT system (3D${}^{2}$YMOX—3-Dimensional DYnamic MOrphology using X-rays) [6] was built for morphological studies of the living animals. The system is highly modular and geometry misalignment occurs in every new acquisition setup. The 3D${}^{2}$YMOX system is discussed further in Section 2.1. To obtain a high quality tomographic reconstruction and to exploit the benefits of a stereo X-ray CT setup, the system needs to be calibrated by estimating the geometric relationship between the X-ray source and the detector pairs with respect to the rotation axis prior to image reconstruction.

Many studies have dealt with single cone-beam X-ray CT system calibration. They include self-calibration methods [7,8,9,10], which calculate the geometry parameters of the acquisition system directly from the acquired radiographs of the target objects, and calibration phantom-based techniques [11,12,13,14]. In the work presented by Parkinson et al. [7], the object orientation parameters were estimated from an iterative, projection matching and reconstruction-based procedure. This was followed by a calculation of the 2D shifts of the rotation axis with reference to the detector coordinate system. In another study, Kingston et al. [8] estimated 3D orientation of the rotation axis and the detector position on the optical axis by iteratively transforming the projection data with respect to the geometry parameters to refine the sharpness of reconstructed CT images. Both methods are, however, computationally expensive as iterative CT reconstruction is required to estimate the geometry parameters.

Several self-calibration techniques effectively reduced calculation cost with the projection-based procedures. For example, Kyungtaek et al. [9] used the projection trajectory of a fixed point to estimate the translation and the vertical tilt of the rotation axis, however, it can only be applied to a parallel 3D geometry. Wang et al. [10] introduced a two-step algorithm to consecutively correct the misalignments caused by vertical translation and tilt, followed by a correction of horizontal translation of the target object in the raw projections. In general, self-calibration with a projection-based procedure is dependent on the object’s orientation and position with respect to its projection’s coordinate system and therefore requires calibration prior to each scan, even without any change of the geometry setup.

Most X-ray CT geometry calibration methods that rely on fiducial markers employ specifically designed phantoms in which the position of the markers is measured accurately using Coordinate Measuring Machines (CMM). For example, Liu et al. [11] introduced a phantom that carried 12 spherical zirconia markers placed on a triple helix glass phantom. Another well designed phantom was presented by Cho et al. and Chetley et al. [12,13] with two rings of evenly placed steel markers on an acrylic cylinder. Efforts have been made to reduce the calibration phantom complexity. For example, Mennessier et al. [14] presented a comprehensive analytical method to compute the cone-beam geometry parameters using a 14-marker phantom. The studies reported a high level of calibration accuracy and reduced misalignment artifacts on the CT volumes.

Only a few studies have been reported on calibrating the geometry of a stereo X-ray CT system, [15,16,17]. Chang et al. [15] presented a method to calibrate a dual-axis tomosynthesis system with an acrylic plate holding non-solid and solid spheres attached in fixed and known grid positions. The reference marker centers are iteratively aligned to those on the calibration radiographs to estimate the detector orientation and the source position. Sawall et al. [16] introduced a phantom-based calibration procedure in which the position of the sphere marker was not known. Its position is estimated by solving a least squares problem given a measured (nominal) system geometry. A genetic optimization algorithm was used to fine-tune the position of the source as well as the detector orientation. However, the method required the rotation stage to be manipulated with under 1 mm accuracy and the nominal system geometry to be well-measured, these requirements are not fulfilled in the 3D${}^{2}$YMOX system. In another study, Allab et al. [17] reported a phantom-based calibration method as an alternative to the box calibration cage, which is usually used in radio stereometric analysis applications as a reference frame to locate the positions of implants or bones in the patients. The calibration procedure required no prior knowledge of the marker positions in the phantom. The geometry parameters and the marker coordinates were estimated by iteratively matching the reconstructed 3D point-cloud of the phantom with its reference model. A default calibration is required to generate the initial reference model for calibration, and yet this is not the case for the 3D${}^{2}$YMOX system as no pre-calibrated information available.

In previous work [18], we presented a LEGO phantom-based calibration technique for a single cone-beam X-ray system. However, the angle between the two systems was not taken into account, it is necessary to have a comprehensive calibration procedure that estimates the relative position of the two cone-beam X-ray CT systems. With the angle between the two systems being known, the benefits of a stereo X-ray CT setup can be exploited such as extending the system field-of-view beyond the physical size of single system detector [19] or enhancing CT reconstruction quality as extra data can be acquired in an acquisition from two orthogonal X-ray systems. By choosing two different source energies, more object detail can be revealed in the CT images. Furthermore, when the stereo geometry is fully calibrated, the two CT volumes obtained from two single systems can also be registered.

In this paper, we introduce a full procedure to calibrate the geometry of a modular stereo cone-beam CT system with a LEGO phantom containing metal markers, strategically placed in the bearing bricks. The LEGO phantom is easy to build and to customize to suit the size of target systems. The positions of the markers in the phantom can be calculated from the dimensions of LEGO bricks at a reliable accuracy as LEGO bricks are molded with dimensional tolerance of 5 μm [20]. Furthermore, our proposed calibration method requires no pre-calibrated geometry information and is capable of calibrating a modular stereo cone-beam X-ray CT system such as the 3D${}^{2}$YMOX system. The paper is structured as follows. Section 2 presents our proposed methodology to build a low-cost calibration phantom using LEGO bricks and metal markers along with a deep learning-based procedure to accurately estimate the bead centers. Section 3 discusses the experiments that were performed to validate our proposed method. Finally, further discussion and conclusions are presented in Section 4.

## 2. Methodology

### 2.1. 3D${}^{2}$YMOX System

**Figure 1:**
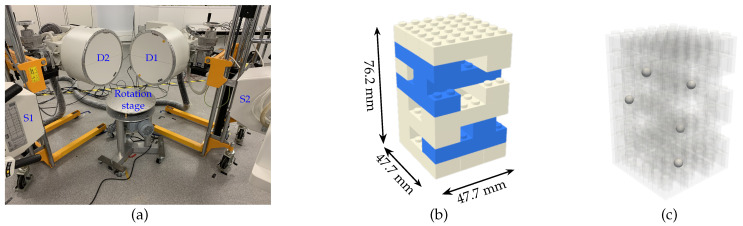
The 3D${}^{2}$YMOX system (**a**), LEGO calibration phantom with embedded metal markers in the blue bricks (**b**) and its transparent view (**c**).

Figure 1a shows the 3D${}^{2}$YMOX system (3-Dimensional DYnamic MOrphology using X-rays system) [21] that is used for morphological and biomechanical research on living animals. The system consists of two X-ray source detector pairs (S1, D1 and S2, S2) and a rotation stage which is mounted on a wheeled tripod (Figure 1a). The sources (S1, S2) are mounted on two ceiling gantries that allow them to be easily positioned in 3D space. The orientation of each source around three principle axes are controlled by side handle bars attached to it. In addition, the two detectors (D1, D2) are put on two trolleys with hydraulic lifts so as to adjust their horizontal and vertical position. Moreover, each detector has a steering wheel that manipulates its orientation in 3D space. Consequently, each device is positioned independently from the others, and therefore, in any new installation, the position and orientation of the source and the detector as well as the position of the stage can change dramatically. With such a setup, it is challenging to align the sources, detectors and rotation stage properly and to accurately measure the geometry. It is, therefore, essential to perform a calibration in order to estimate the system geometry as accurately as possible.

### 2.2. LEGO Calibration Phantom

Phantom-based calibration methods make use of marker (metal bead) positions in the measured X-ray projections to estimate the geometry parameters. To facilitate the extraction of markers from the X-ray projections, the phantom must be built so as to maximize the contrast between LEGO structure and the metal markers in the radiographs. Steel markers with a diameter of (4950±10) μm are embedded in the hollow cylinders of the bricks, by pushing the LEGO bricks on a flat surface to press the metal markers exactly one diameter deep into the cylinders. These marker bearing bricks are then placed such that no two markers are within the same vertical brick layer in the phantom (blue the LEGO bricks in Figure 1). This design avoids overlapping markers in the projections.

Moreover, the markers are placed close to the phantom’s facets to maximize the covering area of their projection trajectories on the detector field-of-view. As studied by Ferrucci et al. [22], the coordinate changes due to geometry misalignments are dependent on the marker positions with respect to the rotation axis. A strategic design of the phantom addresses these coordinate deviations in the misaligned geometry. The phantom dimensions and the number of markers can be customized to suit the size of the system field-of-view. The dimensions of the LEGO bricks and the metal markers were measured by an electronic caliper with 10 μm accuracy. The 3D positions of the metal markers in the phantom are calculated relatively to the dimensions of a single LEGO brick.

According to the company disclosure [20], the LEGO bricks are molded with dimensional tolerance of 5 μm. With a detector pixel size of 142 μm and a magnification factor of ≈1.3 [6], the effective voxel size of the 3D${}^{2}$YMOX system is around 100 μm. The dimensional tolerance of the LEGO bricks is, therefore, ≈20 times smaller than the effective voxel size of the target system. With current 292 × 292 mm image intensifier screens of the 3D${}^{2}$YMOX system, the LEGO calibration phantom was built with only eight-brick-layers tall (76.2 mm), width of 47.7 mm (single 6×2-brick width), and five metal markers. This structure avoids accumulated dimensional errors of the bricks horizontally. Vertical accumulated tolerance is still below the effective voxel size of the 3D${}^{2}$YMOX system. Two identical calibration phantoms were built to validate the feasibility to reproduce the LEGO phantom and to study the effect of the dimensional tolerance on the geometry calibration of a real stereo X-ray CT system. In addition to the calibration phantoms, a test phantom was built from the LEGO bricks and metal markers with a different structure and size from the calibration phantom for evaluating CT quality as well as calibration accuracy.

### 2.3. Stereo Geometry Parameters

The geometry of a stereo X-ray CT system can be calibrated separately for each single-source system; however, the stereo angle α between the two systems is not estimated in this procedure. A comprehensive calibration procedure is needed to fully estimate the geometric parameters of a dual source cone-beam X-ray CT setup.

**Figure 2:**
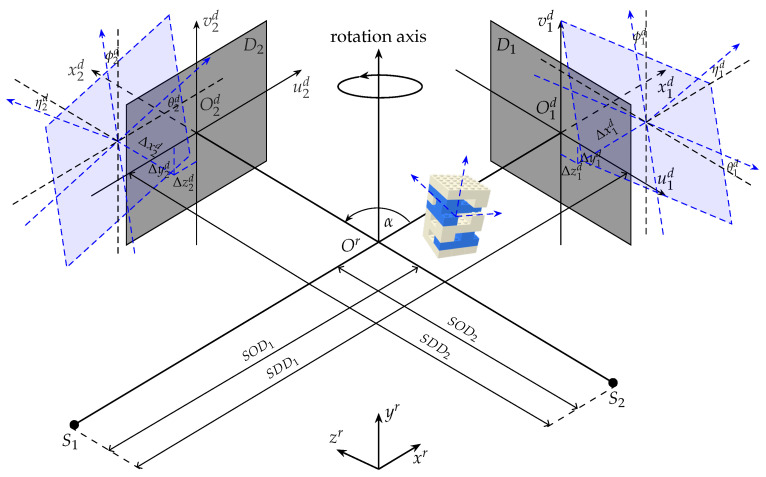
A stereo cone-beam geometry of an X-ray CT system.

Figure 2 shows an aligned stereo cone-beam geometry in black plot with two perpendicular source-detector pairs with reference to their projection axes, hereafter referred to as S1,D1 and S2,D2. The sources and the detectors are stationary during acquisition while the object is rotated around the rotation axis. The distances from either source to its corresponding detector (SDD) and the rotation axis (SOD) are known for each acquisition. Two 3D coordinate systems $O^{r}x^{r}y^{r}z^{r}$ and either $O_{1}^{d}x_{1}^{d}u_{1}^{d}v_{1}^{d}$ or $O_{2}^{d}x_{2}^{d}u_{2}^{d}v_{2}^{d}$ that originate at the center of rotation and the center of the detector panel, are aligned.

To calibrate the system geometry, position and orientation of the calibration phantom need to be defined accurately, for which, they are described by six DOF with respect to the $O^{r}x^{r}y^{r}z^{r}$ including three distance coordinates $\left\{\Delta x^{o},\Delta y^{o},\Delta z^{o}\right\}$ and three orientation angles roll $\theta^{o}$, yaw $\phi^{o}$, pitch $\eta^{o}$ about $\left(x^{r},y^{r},z^{r}\right)$ axis, respectively. The position and the orientation of each detector are defined by six DOF $\left\{\Delta x_{i}^{d},\Delta y_{i}^{d},\Delta z_{i}^{d},\theta_{i}^{d},\phi_{i}^{d},\eta_{i}^{d}\right\}$ with respect to the $O_{i}^{d}x_{i}^{d}u_{i}^{d}v_{i}^{d}$, i={1,2}. The misaligned detectors and corresponding geometry parameters are demonstrated in Figure 2, blue plot.

The distance from the sources to the rotation axis and to the detectors are measured after a new acquisition setup. The measurement uncertainties can be modelled by two more parameters $\Delta sod_{1}$ and $\Delta sod_{2}$. The angle between the two optical axes of the two systems is parameterized by stereo angle α. The stereo cone-beam geometry setup is therefore described by 21 DOF ($\beta =\{\Delta x^{o},\Delta y^{o},\Delta z^{o},\theta^{o},\phi^{o},\eta^{o},\Delta sod_{i},\alpha ,\Delta x_{i}^{d},\Delta y_{i}^{d},\Delta z_{i}^{d},\theta_{i}^{d},\phi_{i}^{d},\eta_{i}^{d}\}$, i={1,2}).

### 2.4. Geometry Calibration

Calibration datasets were acquired with the stereo cone-beam system and the technique presented in [18] was followed to extract the centers of the markers from the radiographs. Calibration methods based on markers make use of the markers’ positions on the detector to estimate the geometry parameters. The reference and the corresponding 2D measured coordinates of marker *k* in projection *n* on the detector plane are denoted as $\left(u_{nk}^{ref},v_{nk}^{ref}\right)$ and $\left(u_{nk}^{mea},v_{nk}^{mea}\right)$, respectively. For every projection angle, the vector that represents the system geometry is transformed with respect to the misalignment parameters. The reference $\left(u_{nk}^{ref},v_{nk}^{ref}\right)$ coordinates are defined as the intersections of the rays from the source through the 3D marker centers $\left(x_{k},y_{k},z_{k}\right)$ with the detector plane.

The measured $\left(u_{nk}^{mea},v_{nk}^{mea}\right)$ coordinates are extracted from the calibration data with template matching technique [23] and fine-tuned using deep learning (BeadNet) [24]. BeadNet is trained from the predefined neural network model Resnet-50 [25] using a simulated dataset containing X-ray bead projections from different geometry configurations.

The geometry parameter set β is estimated by the interior point optimization [26] of the calibration cost function that is the total Euclidean distance between the reference $\left(u_{nk}^{ref},v_{nk}^{ref}\right)$ and the measured $\left(u_{nk}^{mea},v_{nk}^{mea}\right)$ coordinates across all projections $p_{n}$(n=1,…,N) and marker centers k=1,…,K with respect to β:

(1) $$\hat{\beta }=\arg\min_{\beta }\left\{\sum_{n=1}^{N}\sum_{k=1}^{K}\left[{\left(u_{nk}^{ref}\left(\beta \right)-u_{nk}^{mea}\right)}^{2}+{\left(v_{nk}^{ref}\left(\beta \right)-v_{nk}^{mea}\right)}^{2}\right]\right\}$$

where $\beta =\{\Delta x^{o},\Delta y^{o},\Delta z^{o},\theta^{o},\phi^{o},\eta^{o},\Delta sod_{i},\alpha ,\Delta x_{i}^{d},\Delta y_{i}^{d},\Delta z_{i}^{d},\theta_{i}^{d},\phi_{i}^{d},\eta_{i}^{d}\}$, i={1,2}. By iteratively adjusting the geometry parameters to align the reference coordinates $\left(u_{nk}^{ref},v_{nk}^{ref}\right)$ to those on the calibration radiographs ($\left(u_{nk}^{mea},v_{nk}^{mea}\right)$), the geometry parameters are estimated. In the experiment with real datasets, all the geometry parameters are initialized to 0 as no prior knowledge of the geometry parameters is available in the 3D${}^{2}$YMOX system. A complete stereo geometry calibration procedure is as follows. The phantom orientation around vertical axis $\phi^{o}$ and its position $\Delta y^{o}$ are estimated first to align the object vertically. Next, the phantom translations $\left\{\Delta x^{o},\Delta y^{o},\Delta z^{o}\right\}$ are calibrated followed by its orientation parameters $\left\{\theta^{o},\phi^{o},\eta^{o}\right\}$. Then, the calibration phantom parameters along with the stereo angle are estimated using both datasets followed by $\Delta sod_{1}$ and the orientation and translation of D1 optimization. Finally, $\Delta sod_{2}$ and D2 geometry parameters are estimated before all 21 DOF are fine-tuned. This whole procedure is iterated until the calibration cost function shown in Equation (1) converges.

The procedure is iterated 30 times for parameter fine-tuning as either cost function residual or parameter updates are less than 10${}^{-6}$ during iterative optimization. The calibration took around 450 s to finish on an Intel(R) Core(TM) i7-6800K CPU 3.40 GHz PC, with six CPU cores multithreading.

## 3. Experiments and Results

### 3.1. Experiment with Simulated Data

The noiseless simulated stereo datasets included a calibration and a test dataset of 61×2 and 600×2 radiographs generated with a calibration phantom and the test phantom, respectively. The ASTRA CAD projector [27] casts the X-ray beams through the CAD models of the phantoms in the stereo cone-beam geometry, which was modified with the geometry parameters, to generate the simulated radiographs of the phantoms. The detectors were simulated as two square flat panels with 2048×2048− pixels resolution and pixel size of 142 μm.

Initializations, ground truth (GT) and estimated stereo geometric parameters with corresponding uncertainties are presented in Table 1. As shown in the table, optical translations (translations along the projection axes) $\Delta x^{d},\Delta sod$ are estimated with errors on the order of several millimeters as the differences are 470 μm, 3.6 mm, 820 μm and 8.2 mm for $\Delta x_{1}^{d},\Delta x_{2}^{d},\Delta sod_{1},\Delta sod_{2}$, respectively. However the other translation parameters including $\Delta x^{o},\Delta y^{o},\Delta z^{o},\Delta y^{d}$, and $\Delta z^{d}$ are estimated with maximum deviation from the ground-truth values of 170 μm. Orientation parameters are calibrated with a precision below 0.1 deg.

The calibration procedure started with the stereo angle α being initialized to 90${}^{\circ }$ and all the other geometry parameters were initialized to 0. SOD and SDD were fixed to the simulated ground-truth values of {779,1123} mm and {783,1141} mm for S1,D1 and S2,D2, respectively. To further evaluate the impact of the calibration errors on the CT reconstruction quality, the geometry of the S1,D1 and S2,D2 systems were modified with the initialized and calibrated geometric parameters prior to the reconstructions of the test phantom and a piglet specimen. A SIRT algorithm was used to reconstruct the datasets with high-performance GPU primitives ASTRA toolbox [28,29].

**Figure 3:**
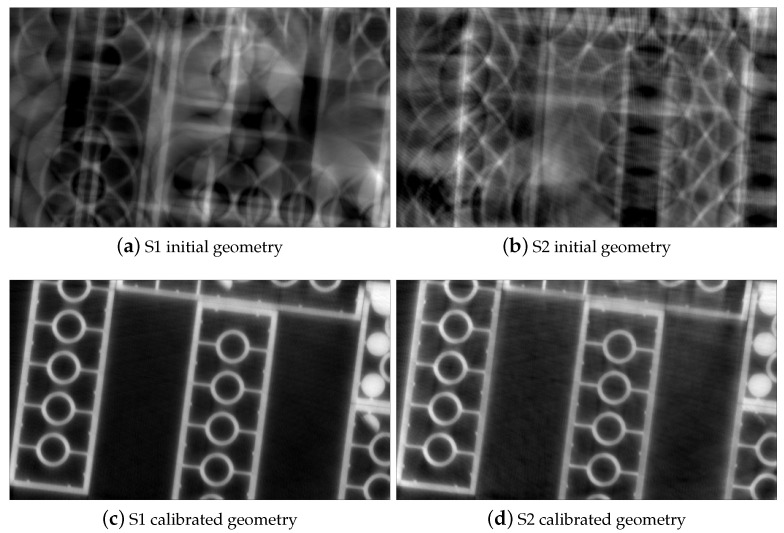
Reconstructions of the test phantom with initial and calibrated stereo geometry parameters for simulated stereo datasets. The LEGO bricks are sharply recovered, misalignment artifacts are eliminated in the CT slices with calibrated geometry (**c**,**d**) compared to without applying misalignment correction (**a**,**b**).

Four transverse images of the reconstructed phantom with S1 and S2 datasets are displayed in Figure 3. Without calibration, the geometry misalignments induce severe blur on the edge of the LEGO bricks Figure 3a,b. After applying the transformation to the geometry vector with estimated parameters, the misalignment artifacts are corrected Figure 3c,d. We obtain sharp and clear LEGO bricks as well as phantom structure. The two slices are also aligned as the stereo angle was accounted for.

### 3.2. Experiments with Real Data

Along with the test phantom, a piglet dataset was used to evaluate quality of the CT images with calibrated geometry. Real X-ray radiographs of the calibration phantoms, the test phantom, and the piglet were acquired by the 3D${}^{2}$YMOX [6] system at a resolution of 2048×2048− pixels.

In this acquisition, the datasets were acquired with two source energies and currents of 57 kV, 40 mA and 59 kV, 40 mA for S1 and S2, respectively. The X-ray tubes were limited to a duration of six seconds of continuous radiation to avoid overloading of the X-ray tube. Four modes of acquisition are available with a maximum of 900 X-ray frames per rotation. With these technical constraints, it is beneficial to incorporate both datasets into a single CT reconstruction to enhance the CT quality in either single or dual energy mode. In these experiments, each of the calibration datasets, the test phantom contains 360 stereo projections while 450 projections of the piglet were acquired from each cone-beam X-ray system for CT reconstruction.

The X-ray image intensifiers in the 3D${}^{2}$YMOX system cause two major geometric distortions, namely pincushion and sigmoidal distortion [30]. The pincushion distortion is the result of the incident X-ray to be detected on a curved input phosphor, while the latter is due to the magnetic interaction of the produced photo-electrons inside the image intensifier. The projection-dependent distortion correction described in [30] was applied to correct for these distortions. Flatfield and log correction were applied to the acquired radiographs to compensate for the different responses in the detectors.

As shown in the simulated experiments, $\Delta sod_{i}$ along with the detector position on the optical axis ($\Delta x_{i}$), i={1,2} are highly correlated and all impact on the magnification of the object projection. Taking into account both parameters induces large redundancy and error in the calibration. Therefore, $\Delta sod_{i}$ is eliminated in the experiments with the real datasets. Only detector displacements along two optical axes $\Delta x_{i}^{d}$, i={1,2} are accounted for calibration.

The stereo geometry was calibrated with 90 radiographs acquired from each single-source system. In these experiments, all the geometry parameters were initialized to 0. SOD and SDD were fixed to the measurements of {1002,1303} mm and {978,1226} mm for S1,D1 and S2,D2, respectively. The procedure was repeated with both calibration phantom datasets and the initializations along with the estimated geometry parameters are shown in Table 2. As can be seen in the table, the detector translation and orientation parameters are calibrated with maximum differences of 3.5 mm and 6.2${}^{\circ }$, respectively. Both calibrations with the two phantoms derive the same value of the stereo angle α.

**Figure 4:**
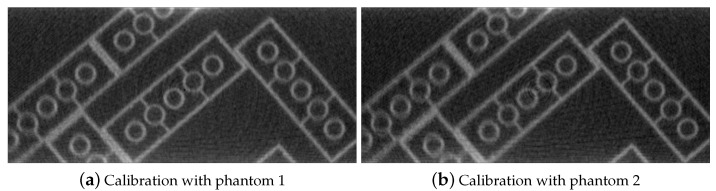
Reconstructions of the test phantom acquired by the 3D${}^{2}$YMOX system after stereo geometry calibration with real calibration phantoms.

To study the impact of these differences of the calibrated parameters with two calibration phantoms on quality of the reconstructed images, a test phantom dataset acquired in the 3D${}^{2}$YMOX system was reconstructed with two sets of calibrated parameters by ASTRA toolbox [28,29] SIRT algorithm. Two CT slices of the test phantom are shown in Figure 4. As shown in the figures, more apparent misalignment artifacts appear in the reconstruction with calibrated geometry by phantom 2 (Figure 4b, lower-left corner), compared to the result with phantom 1 (Figure 4a).

**Figure 5:**
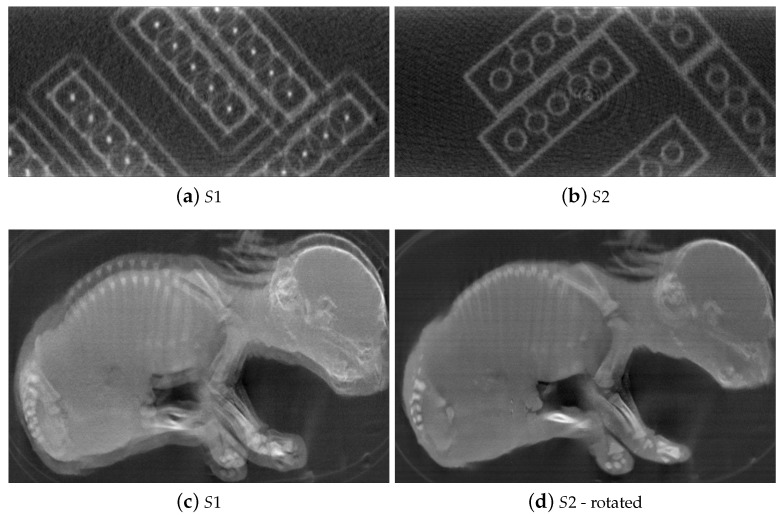
Reconstructions of the test phantom and the piglet with real datasets acquired by the 3D${}^{2}$YMOX system and the initializations of the stereo geometry parameters. The misalignment artifacts is less severe in the reconstruction with S2,D2 datasets (**b**,**d**) compared to (**a**,**c**) due to the initializations of the geometric parameters turning out to be more precise.

Figure 5 shows four CT slices from the reconstructed volumes of the test phantom (a,b) and the piglet (c,d) stereo dataset without geometry calibration. The reconstructed slices a of the test phantom Figure 5 with dataset from S1 is in a different orientation compared to the slice from the S2 dataset (Figure 5b). In Figure 5d, the CT slice of the piglet specimen with the S2 dataset was rotated to a similar orientation as in Figure 5c for a better visualization. This orientation difference is mainly due to the uncalibrated stereo angle. Moreover, without compensating for the geometry misalignment, the LEGO bricks and the piglet’s internal structure are blurry due to the misalignment artifacts. The effect of misalignment is more severe in the reconstruction with the S1 datasets as shown in Figure 5a,c compared to the CT slices from S2 (Figure 5b,d) due to less accurate measurements of $SOD_{1}$ and $SDD_{1}$.

**Figure 6:**
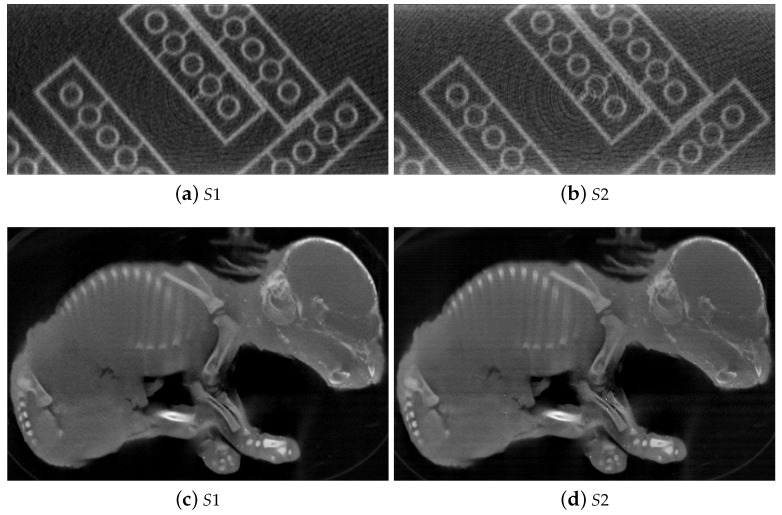
Reconstructions of the test phantom (**a**,**b**) and the piglet (**c**,**d**) datasets acquired by the 3D${}^{2}$YMOX system with calibrated geometry. With geometry correction, the LEGO bricks and the piglet structure are clearly visible in the CT slices.

With a corrected geometry, the misalignment artifacts are significantly suppressed revealing a clear image of the LEGO bricks and the piglet skeleton as shown in Figure 6. As the stereo angle was taken into account, the CT slices obtained with the datasets from S1 and S2 are aligned in the same orientation. This result suggests a possibility to have dual-energy view of an object acquired with the 3D${}^{2}$YMOX system.

**Figure 7:**
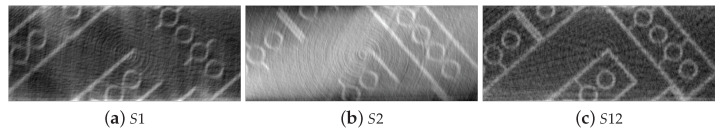
Reconstructions of the test phantom datasets from single (**a**,**b**) and dual (**c**) X-ray source of the 3D${}^{2}$YMOX system with 60${}^{\circ }$ missing wedge of either single system. The reconstruction of dual-source dataset (**c**) show that the missing wedge artifact can be corrected by incorporating both single datasets into the stereo reconstruction.

To study the benefit of the dual-source acquisition, three reconstructions of the test phantom were done with two single-source and a dual-source datasets. The single source datasets are a subset of the full rotation dataset with a missing angular range of 60${}^{\circ }$. Two single-source datasets are concatenated to generate a dual-source dataset as if it was acquired with a stereo angle, which is a sequence of the projection angles of S1,D1 and their shifts by the stereo angle α. The calibrated parameters of both systems were then used for the reconstruction of the dual-source dataset. As shown in Figure 7, the CT slices reconstructed with the single source datasets (a, b) suffer from missing wedge artifacts as the LEGO bricks were only partly reconstructed in both slices, while in the dual-source slice (c) the LEGO bricks are fully reconstructed. This experiment demonstrated that, with a calibrated stereo geometry of the 3D2YMOX system, it is possible to reconstruct two datasets acquired simultaneously and/or in dual-energy mode from the two single cone-beam X-ray systems.

## 4. Discussions and Conclusions

In this work, we presented a comprehensive method to calibrate the 3D${}^{2}$YMOX system, which is a highly modular stereo X-ray CT system, with a LEGO phantom. The simulation experiments demonstrated that a LEGO phantom can be used to accurately calibrate the geometry of a stereo X-ray CT system, with exception of the optical translation parameters. This can be explained by a high correlation between these two parameters due to which the errors cancel each other out in the reconstruction. This was also verified in the experiments with real datasets, translation of the rotation center along the optical axis was excluded from the calibration. The reconstructions of the piglet data shows significantly misalignment artifact-free after stereo geometry alignment.

When the stereo angle is accounted for in the reconstruction, the two CT volumes obtained from the individual X-ray systems are aligned, and the two dataset acquired with each X-ray cone-beam system can be combined for a stereo reconstruction, opening up the possibility of a dual energy and/or a faster scan. Experiments with two real LEGO phantom dataset demonstrated that our proposed method can be applied to practical dual X-ray CT systems. Further study on the difference between the geometry parameters estimated with two identical calibration phantom datasets and its impact on the reconstruction quality need to be done.

In the experiment with the real datasets, the differences in CT reconstruction quality between the datasets acquired with S1 and S2 can be explained by the fact that the two single cone-beam X-ray systems are not identical. The X-ray projections as well as the flatfield images acquired from the two systems differ in terms of intensity and noise level, and therefore result in unequal reconstruction quality and contrast. A further study needs to be done on acquisition settings in terms of hardware and software configurations to optimize the CT reconstruction quality.

In conclusion, the proposed LEGO calibration procedure can be a valuable solution to calibrate the stereo geometry of dual cone-beam X-ray CT systems. In future work, we aim at fully evaluating the CT reconstruction quality with the calibrated stereo geometry parameters. A further study on quantifying calibration accuracy in terms of voxel resolution also needs to be done.

## Figures and Tables

**Table 1:** Calibration errors of the geometric parameters for a simulated stereo X-ray CT system. Optical translations $\Delta x^{d}$ and Δsod are estimated with errors on the order of millimeters (red) due to high correlation between them.

	$\Delta x^{\circ }$	$\Delta y^{\circ }$	$\Delta z^{\circ }$	$\Delta x_{1}^{d}$	$\Delta y_{1}^{d}$	$\Delta z_{1}^{d}$	$\Delta {\mathrm{sod}}_{1}$	$\Delta x_{2}^{d}$	$\Delta y_{2}^{d}$	$\Delta z_{2}^{d}$	$\Delta {\mathrm{sod}}_{2}$
Init. (mm)	0.00	0.00	0.00	0.00	0.00	0.00	0.00	0.00	0.00	0.00	0.00
GT (mm)	−7.96	12.6	−12.6	19.7	−18.5	−10.1	7.21	−10.2	−17.8	14.0	11.9
Err. (μm)	14	120	2	470	36	2	820	3600	170	1	8200
	$\theta^{o}$	$\phi^{o}$	$\eta^{o}$	α	$\theta_{1}^{d}$	$\phi_{1}^{d}$	$\eta_{1}^{d}$	$\theta_{2}^{d}$	$\phi_{2}^{d}$	$\eta_{2}^{d}$	
Init. (${}^{\circ }$)	0.00	0.00	0.00	90.0	0.00	0.00	0.00	0.00	0.00	0.00	
GT (${}^{\circ }$)	4.08	187	4.29	94.1	3.81	2.02	2.08	4.24	2.01	0.67	
Err. (deg)	0.004	0.015	0.010	0.008	0.006	0.031	0.021	0.007	0.098	0.070	

**Table 2:** Calibrated geometry parameters of the 3D${}^{2}$YMOX system with two identical calibration phantoms.

(mm)	$\Delta x^{o}$	$\Delta y^{o}$	$\Delta z^{o}$	$\Delta x_{1}^{d}$	$\Delta y_{1}^{d}$	$\Delta z_{1}^{d}$	$\Delta x_{2}^{d}$	$\Delta y_{2}^{d}$	$\Delta z_{2}^{d}$	
Inits.	0.00	0.00	0.00	0.00	0.00	0.00	0.00	0.00	0.00	
Phantom 1	−16.6	66.1	−10.1	−5.31	−31.6	−3.35	−4.67	−30.63	−0.06	
Phantom 2	16.7	65.4	−22.3	−1.85	−30.8	−3.61	−6.08	−29.6	−0.05	
(deg)	$\theta^{o}$	$\phi^{o}$	$\eta^{o}$	α	$\theta_{1}^{d}$	$\phi_{1}^{d}$	$\eta_{1}^{d}$	$\theta_{2}^{d}$	$\phi_{2}^{d}$	$\eta_{2}^{d}$
Inits.	0.00	0.00	0.00	0.00	0.00	0.00	0.00	0.00	0.00	0.00
Phantom 1	0.401	−28.9	0.612	89.6	−0.049	−3.41	−0.89	0.459	−2.00	−1.12
Phantom 2	0.118	42.6	0.372	89.6	−0.424	2.79	−4.90	0.441	−1.44	−1.02

## Data Availability

Restrictions apply to the availability of these data. Data was obtained from imec-Vision Lab and are available from the authors with the permission of imec-Vision Lab.
